# Sex- and Age-Based Disparities in Public Access Defibrillation, Bystander Cardiopulmonary Resuscitation, and Neurological Outcome in Cardiac Arrest

**DOI:** 10.1001/jamanetworkopen.2023.21783

**Published:** 2023-07-05

**Authors:** Masanobu Ishii, Kenichi Tsujita, Tomohisa Seki, Masafumi Okada, Kazumi Kubota, Kenichi Matsushita, Koichi Kaikita, Naohiro Yonemoto, Yoshio Tahara, Takanori Ikeda

**Affiliations:** 1 Department of Cardiovascular Medicine, Graduate School of Medical Sciences, Kumamoto University, Kumamoto, Japan; 2 Department of Healthcare Information Management, The University of Tokyo Hospital, Tokyo, Japan; 3 Integrated Evidence Generation, Medical Affairs and Pharmacovigilance, Bayer Yakuhin Ltd, Tokyo, Japan; 4 Division of Cardiovascular Medicine and Nephrology, Department of Internal Medicine, Faculty of Medicine, University of Miyazaki, Kiyotake, Miyazaki, Japan; 5 Japanese Circulation Society with Resuscitation Science Study (JCS-ReSS) Group, Tokyo, Japan

## Abstract

**Importance:**

Younger females with out-of-hospital cardiac arrest (OHCA) in public locations have less likelihood to receive public access defibrillation and bystander cardiopulmonary resuscitation (CPR). However, the association between age- and sex-based disparities and neurological outcomes remains underexamined.

**Objective:**

To investigate the association between sex and age and the rate of receiving bystander CPR, automated external defibrillator defibrillation, and neurological outcomes in patients with OHCA.

**Design, Setting, and Participants:**

This cohort study used the All-Japan Utstein Registry, a prospective, population-based, nationwide database in Japan containing data on 1 930 273 patients with OHCA between January 1, 2005, and December 31, 2020. The cohort comprised patients with OHCA of cardiac origin that was witnessed by citizens and treated by emergency medical service personnel. The data were analyzed from September 3, 2022, to May 5, 2023.

**Exposure:**

Sex and age.

**Main Outcomes and Measures:**

The primary outcome was favorable neurological outcome at 30 days after an OHCA. Favorable neurological outcome was defined as a Cerebral Performance Category score of 1 (indicating good cerebral performance) or 2 (indicating moderate cerebral disability). The secondary outcomes were the rates of receiving public access defibrillation and bystander CPR.

**Results:**

The 354 409 included patients who experienced bystander-witnessed OHCA of cardiac origin had a median (IQR) age of 78 (67-86) years and 136 520 were females (38.5%). The rate of receiving public access defibrillation was higher in males than females (3.2% vs 1.5%; *P* < .001). Stratified by age, age- and sex-based disparities in prehospital lifesaving interventions by bystanders and in neurological outcomes were observed. Although younger females had a lower rate of receiving public access defibrillation and bystander CPR than males, younger females had a higher favorable neurological outcome compared with males of the same age (odds ratio [OR], 1.19; 95% CI, 1.08-1.31). In younger females with OHCA that was witnessed by nonfamily bystanders, receiving public access defibrillation (OR, 3.51; 95% CI, 2.34-5.27) or bystander CPR (OR, 1.62; 95% CI, 1.20-2.22) was associated with a favorable neurological outcome.

**Conclusions and Relevance:**

Results of this study suggest a pattern of significant sex- and age-based differences in bystander CPR, public access defibrillation, and neurological outcomes in Japan. Improvement in neurological outcomes in patients with OHCA, especially younger females, was associated with increased use of public access defibrillation and bystander CPR.

## Key Points

**Question:**

Are age- and sex-based disparities in prehospital lifesaving interventions associated with neurological outcomes in patients who experienced out-of-hospital cardiac arrest (OHCA)?

**Findings:**

In this cohort study of 354 409 patients with OHCA, sex-based disparities in public access defibrillation, bystander cardiopulmonary resuscitation, and neurological outcomes were found from childhood to older age (≥75 years). Although younger females had a higher likelihood for favorable neurological outcome after adjustment of demographic characteristics and prehospital factors, younger females were less likely than their male counterparts to receive public access defibrillation and bystander cardiopulmonary resuscitation.

**Meaning:**

Findings of this study suggest that improvement in the provision of basic lifesaving interventions was associated with higher rates of favorable neurological outcomes among patients experiencing OHCA, particularly younger females.

## Teaser

This cohort study evaluates the risks and outcomes of lifesaving interventions, including cardiopulmonary resuscitation (CPR), provided in public to individuals who experienced out-of-hospital cardiac arrest in Japan.

## Introduction

Sudden cardiac death is one of the leading causes of mortality and is a public health issue that should be resolved.^1,2,3^ Early attempts at bystander cardiopulmonary resuscitation (CPR) and defibrillation using an automated external defibrillator (AED) play a crucial role in the chain of survival for out-of-hospital cardiac arrest (OHCA).^4^ In Japan, the number of public access AEDs and the proportion of patients with OHCA receiving defibrillation using public access AEDs have increased from 1.1% in 2005 to 16.5% in 2013.^5^ Furthermore, reports showed that patients with OHCA who received defibrillation with public access AED had better survival and favorable neurological outcomes.^6,7^

Although AEDs have become widely available in public settings, the issue of sex-based disparities in AED use for patients with OHCA has been raised. Previous studies found that females with OHCA aged 18 to 64 years were less likely to receive bystander CPR in public locations and, when witnessed by a nonfamily member, were less likely to receive bystander CPR regardless of age.^8^ Additionally, females had worse neurological outcomes compared with males in public locations but not in residential locations.^8^ Regarding public access AED, studies have reported a lower rate of AED pad application by laypersons in public locations in young and middle-aged females compared with males of the same age.^9,10^ This accumulating evidence suggests that public basic life-support interventions are underused for females, and the association between this issue and neurological outcomes has not been fully examined.

In this study, we aimed to investigate the association between sex and age and the rate of receiving bystander CPR, AED defibrillation, and neurological outcomes in patients with OHCA. We used the nationwide database All-Japan Utstein Registry (UMIN-CTR Clinical Trials, identifier UMIN000009918) of the Fire and Disaster Management Agency (FDMA).

## Methods

The Institutional Review Board of Kumamoto University Hospital approved the study protocol and deemed it exempt from the informed consent requirement because all of the data used were deidentified. This cohort study was conducted according to the Declaration of Helsinki.^11^ A resuscitation science subcommittee of the Japanese Circulation Society obtained the registry data after approval of the prescribed government legal procedures and provided clean, anonymized data. We followed the Strengthening the Reporting of Observational Studies in Epidemiology (STROBE) reporting guideline.

### Study Design, Population, and Settings

This retrospective cohort study obtained data from the All-Japan Utstein Registry, a prospective, population-based, nationwide registry of all patients with OHCA in Japan, as previously described.^5,6,8,12,13,14^ Data are recorded in the registry using an internationally standardized Utstein template.^15,16^ Between January 1, 2005, and December 31, 2020, a total of 1 930 273 patients who experienced OHCA in Japan were entered into the registry. The registry does not include a race and ethnicity variable.

After exclusion of patients who did not meet the inclusion criteria, 354 409 patients with OHCA of cardiac origin witnessed by citizens and then treated by emergency medical service (EMS) personnel were selected (eFigure 1 in Supplement 1). The exclusion criteria were as follows: no attempted resuscitation (n = 42 810); not witnessed by bystanders (n = 1 111 931); witnessed by EMS personnel (n = 151 814); unknown witness status (n = 6236); unknown bystander CPR status (n = 651); rescue-breathing-only CPR (n = 5097); missing age data (n = 19); unknown outcome data (n = 21); and OHCA of noncardiac origin (n = 257 285), which was clinically ascertained by the responsible medical practitioners in conjunction with the EMS personnel. Noncardiac origin included respiratory diseases (n = 62 807); cerebrovascular disease (n = 21 470); malignant tumors (n = 22 672); and external factors (n = 62 479), including hanging and asphyxia, traffic trauma (n = 10 026), drowning (n = 2076), drug overdose (n = 216), anaphylaxis (n = 158), hypothermia (n = 127), or any other noncardiac factors (n = 75 254).

Patients were categorized according to sex (male or female) and age. Age was grouped either in 5-year bands (ie, 0-4 years to ≥85 years) or as follows: childhood (0-14 years), reproductive (15-49 years), middle to young-old (50-74 years), or older (≥75 years).^9^

Cardiac arrest was assumed to be of cardiac origin (unless it was due to cerebrovascular disease; respiratory diseases; malignant tumors; or external factors, such as trauma, hanging, drowning, drug overdose, asphyxiation, or other noncardiac origin) and was clinically diagnosed by physicians in collaboration with EMS personnel. Details of the EMS system, which is provided 24 hours every day; CPR training for the general public; and dissemination of public access AEDs in Japan have been described elsewhere.^5,6,8,17^

### Variables

Since January 2005, OHCA data in the Utstein format^15,16^ have been prospectively collected and checked by the FDMA for missing or duplicate information. When the data form was incomplete, the FDMA asked the responsible fire station to complete it. From the registry, the following variables were obtained: age, sex, origin of cardiac arrest (cardiac or noncardiac), whether resuscitation was attempted by EMS personnel, date of OHCA event, prefectures with responsible municipalities (47 prefectures), status of witnessed cardiac arrest (citizen witnessed, EMS witnessed, unwitnessed, or unknown), relationship of bystander to patient (family member or nonfamily bystander), whether bystander CPR was assisted by EMS dispatcher, type of bystander-initiated CPR (chest compressions only, chest compressions with rescue breathing, or none), whether public access defibrillation was performed by bystander, whether advanced airway management was performed, whether epinephrine was administered, first documented cardiac arrest rhythm (ventricular fibrillation [VF], pulseless ventricular tachycardia [VT], pulseless electrical activity, asystole, or other), return of spontaneous circulation status of the patient until arrival at hospital, response time (time between patient collapse and initiation of bystander CPR, time between patient collapse and arrival of EMS, or time between emergency call and arrival of EMS), 30-day survival, and 30-day Cerebral Performance Category (CPC) score.

### Outcomes 

The primary outcome was a favorable neurological outcome, defined as a CPC score of 1 (indicating good cerebral performance) or 2 (indicating moderate cerebral disability),^15,16^ in patients at 30 days after an OHCA. The neurological status of patients was assessed by the attending physician 30 days after the return of spontaneous circulation, and the data were completed through a follow-up interview of the attending physician by EMS personnel. The secondary outcomes were the rates of receiving public access defibrillation and bystander CPR in patients with OHCA.

### Statistical Analysis

Data were expressed as medians and IQRs for continuous variables and as numbers and proportions for categorical variables. As appropriate, group comparisons were performed using the Mann-Whitney test for continuous variables and the χ^2^ test or Fisher exact test for categorical variables. A standardized mean difference (SMD) was calculated, and values greater than 0.10 indicated a substantial imbalance.^18^ Logistic regression with mixed effects accounting for the variety of municipalities as a random intercept was performed to compute odds ratios (ORs) and 95% CIs as estimates of females for the study outcomes. In a multivariable analysis, EMS-dispatcher assistance, family member (or nonfamily bystander), time of emergency call, bystander CPR, AED defibrillation, advanced airway management, first documented cardiac arrest rhythm, epinephrine administration, and year of OHCA were adjusted. An interaction test was performed using the variables obtained by multiplying sex and age categories as the interaction term to assess the effect modification by age category.

Sensitivity analysis was performed to assess the association of young female sex with neurological outcomes in patients with OHCA witnessed by bystanders. Additionally, the implications of public access defibrillation and bystander CPR for favorable neurological outcomes in female patients when witnessed by bystanders was assessed using the mixed-effects logistic regression model, and an interaction test was performed using the variables obtained by multiplying basic lifesaving intervention (public access defibrillation or bystander CPR) and age category as interaction term. To confirm the results’ robustness, inverse probability of treatment weighting (IPTW) mixed-effects logistic regression analysis was performed as a sensitivity analysis for favorable neurological outcomes. The estimated probability of female sex was calculated by applying a logistic regression model, using all clinical variables as mentioned previously, and was used as weights for the IPTW models.

A 2-sided *P* < .05 was considered to be statistically significant. All statistical analyses were performed from September 3, 2022, to May 5, 2023, using R, version 4.0.5 (R Foundation for Statistical Computing).^19^

## Results

### Patient Characteristics

From 2005 to 2020, 1 930 273 patients with OHCA were confirmed in Japan, and 354 409 patients who experienced OHCA of cardiac origin that was witnessed by citizens were analyzed in this study (eFigure 1 in Supplement 1). Patient characteristics are summarized in the Table and eTable 1 in Supplement 1. Of these patients, 136 520 were females (38.5%) and 217 889 were males (61.5%) with a median (IQR) age of 78 (67-86) years.

**Table.** Baseline Characteristics of Patients With Out-of-Hospital Cardiac Arrest by Sex

Variable	Patients, No. (%)			Standardized mean difference	*P* value
	Overall	Male	Female		
No. of patients	354 409 (100)	217 889 (61.5)	136 520 (38.5)	NA	NA
Age, median (IQR), y	78 (67-86)	74 (63-83)	84 (74-90)	−0.57	<.001
Relationship of bystander to patient					
Family member	226 358 (64)	142 865 (66)	83 493 (61)	0.09	<.001
EMS-dispatcher–assisted bystander CPR	170 418 (48)	103 526 (48)	66 892 (49)	−0.03	<.001
Type of bystander-initiated CPR					
Chest compressions only	141 450 (40)	85 245 (39)	56 205 (41)	0.07	<.001
Chest compressions with rescue breathing	39 492 (11)	21 855 (10)	17 637 (13)		
None	173 467 (49)	110 789 (51)	62 678 (46)		
Public access defibrillation performed by bystander	9033 (3)	6951 (3)	2082 (2)	0.11	<.001
CPR protocol based on Japan Resuscitation Council guidelines					
2005	117 130 (33)	73 170 (34)	43 960 (32)	−0.02	<.001
2010	115 999 (33)	70 649 (32)	45 350 (33)		
2015	121 280 (34)	74 070 (34)	47 210 (35)		
Time between patient collapse and initiation of bystander CPR, median (IQR), min	2.0 (0.0-5.0)	2.0 (0.0-5.0)	1.0 (0.0-5.0)	0.07	<.001
Missing data	4536 (1)	2772 (1)	1764 (1)		
Time between patient collapse and arrival of EMS, median (IQR), min	9.0 (6.0-13.0)	9.0 (6.0-13.0)	9.0 (6.0-13.0)	0	.002
Missing data	8058 (2)	4760 (2)	3298 (2)		
Time between emergency call and arrival of EMS, median (IQR), min	7.0 (6.0-9.0)	7.0 (6.0-9.0)	7.0 (6.0-9.0)	0.02	.07
Missing data	344 (0)	224 (0)	120 (0)		
Advanced airway management	163 639 (48)	102 904 (49)	60 735 (46)	0.06	<.001
Missing data	11 305 (3)	6830 (3)	4475 (3)		
Epinephrine administration	85 353 (24)	56 059 (26)	29 294 (21)	0.10	<.001
First documented cardiac arrest rhythm					
VF or pulseless VT	72 627 (20)	57 916 (27)	14 711 (11)	−0.38	<.001
PEA	114 235 (32)	66 070 (30)	48 165 (35)		
Asystole	160 076 (45)	89 221 (41)	70 855 (52)		
Other^a^	7471 (2)	4682 (2)	2789 (2)		

Abbreviations: CPR, cardiopulmonary resuscitation; EMS, emergency medical service; NA, not applicable; PEA, pulseless electrical activity; VF, ventricular fibrillation; VT, ventricular tachycardia.

^a^ Included sinus rhythm, atrial fibrillation, and others.

There was no meaningful imbalance between females and males regarding the relationship between bystanders and patients, rate of receiving EMS-dispatcher–assisted CPR, type of bystander-initiated CPR, advanced airway management, epinephrine administration, and response time (Table). In contrast, the distribution of the first documented cardiac arrest rhythm varied: an initial shockable arrest rhythm (VF or pulseless VT) was observed more frequently in males than in females (27% vs 11%; SMD, −0.38), whereas an initial nonshockable arrest rhythm was less frequent in males than females (30% vs 35%; SMD, −0.38). To estimate the initial cardiac arrest rhythm in patients with OHCA, we investigated the first documented cardiac arrest rhythm in patients with OHCA without bystander CPR. As shown in eFigure 2 in Supplement 1, VF or pulseless VT was less frequent in females 25 years or older compared with their male counterparts. On the other hand, there was no statistically significant sex difference among those 24 years or younger.

### Sex- and Age-Based Differences in Receiving Public Access Defibrillation and Bystander CPR

**Figure 1.**
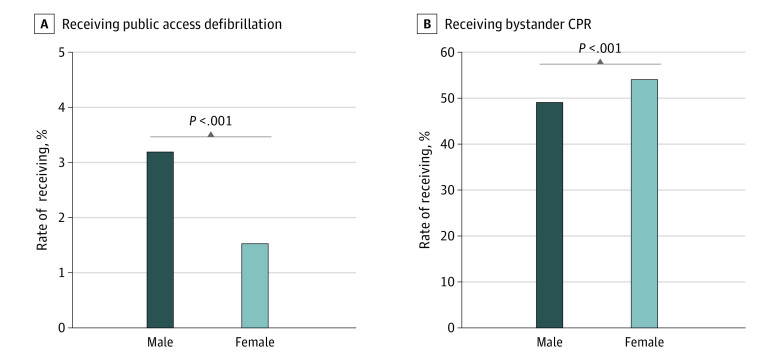
Sex-Based Difference in the Rate of Receiving Public Access Defibrillation and Bystander Cardiopulmonary Resuscitation (CPR) in Patients With Bystander-Witnessed Out-of-Hospital Cardiac Arrest

**Figure 2.**
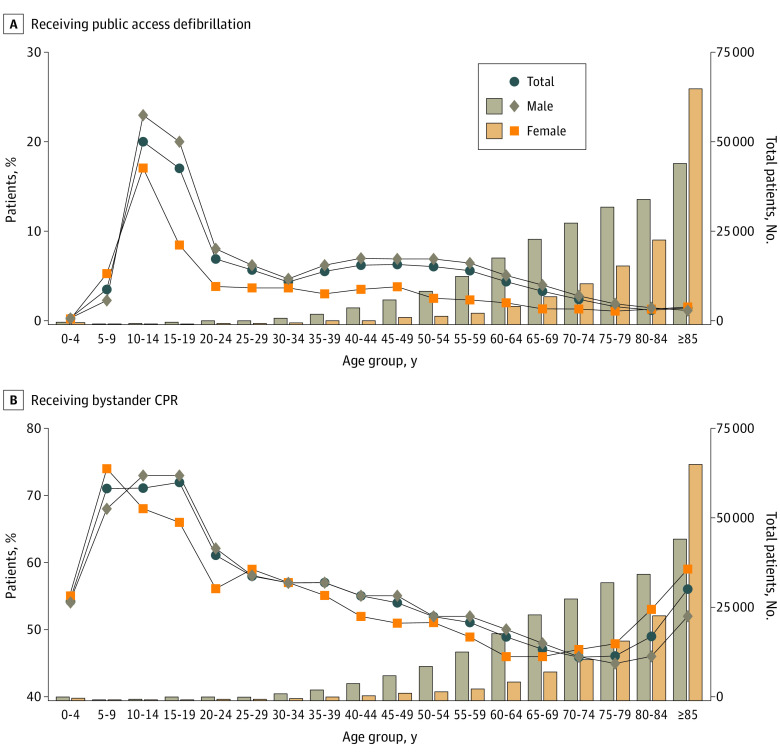
Sex- and Age-Based Differences in Rate of Receiving Public Access Defibrillation and Bystander Cardiopulmonary Resuscitation (CPR) in Patients With Bystander-Witnessed Out-of-Hospital Cardiac Arrest Line graphs show the receipt rate, and bar graphs show the number of patients.

As shown in Figure 1, the rate of receiving public access defibrillation was significantly higher in males than in females (3.2% vs 1.5%; *P* < .001), whereas the rate of receiving bystander CPR was significantly lower in males than females (49.2% vs 54.1%; *P* < .001). Stratified by age category, the rate of receiving public access defibrillation was significantly higher in males than females in the reproductive (7.0% vs 3.8%; *P* < .001), middle to young-old (4.5% vs 1.6%; *P* < .001), and older (1.5% vs 1.4%; *P* = .05) age groups but was not significant in childhood (Figure 2A). The rate of receiving bystander CPR was significantly higher in males than females in the reproductive (56.8% vs 53.5%; *P* < .001) and middle to young-old (48.8% vs 47.0%; *P* < .001) age groups (Figure 2B). In contrast, the rate was significantly lower in males than females in the older age group (48.1% vs 56.1%; *P* < .001) (Figure 2). Furthermore, females in the reproductive age group had significantly lower rates of receiving public access defibrillation (3.8% vs 7.0%; *P* < .001) and bystander CPR (53.5% vs 56.8%; *P* < .001) compared with males.

eFigure 3 in Supplement 1 shows temporal patterns of annual increase in the rates of receiving public access defibrillation and bystander CPR. In the total study population, the rate of receiving public access defibrillation was lower in females than males, whereas the rate of receiving bystander CPR was higher in females than males (eFigure 3A in Supplement 1). In the reproductive age group, sex-based disparities were more evident in temporal pattern of receiving public access defibrillation (eFigure 3B in Supplement 1). However, no sex-based disparities were evident in the pattern when limited to patients with OHCA who had an initial shockable cardiac arrest rhythm (eFigure 3C in Supplement 1).

### Sex- and Age-Based Differences in Neurological Outcomes 30 Days After Cardiac Arrest

**Figure 3.**
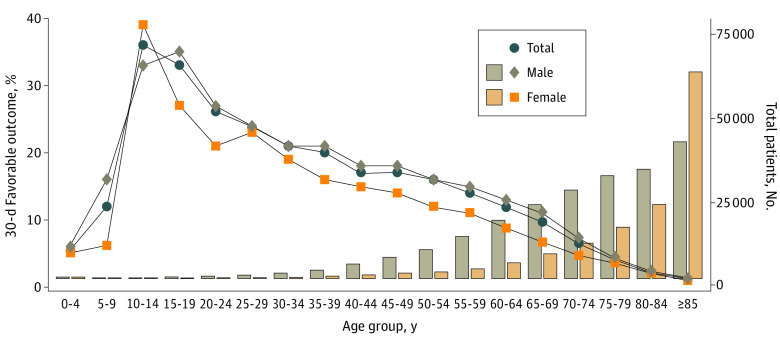
Sex- and Age-Based Differences in Favorable Neurological Outcomes in the Overall Population of Patients With Bystander-Witnessed Out-of-Hospital Cardiac Arrest Line graphs show the rate of 30-day favorable neurological outcomes. Bar graphs show the number of patients.

**Figure 4.**
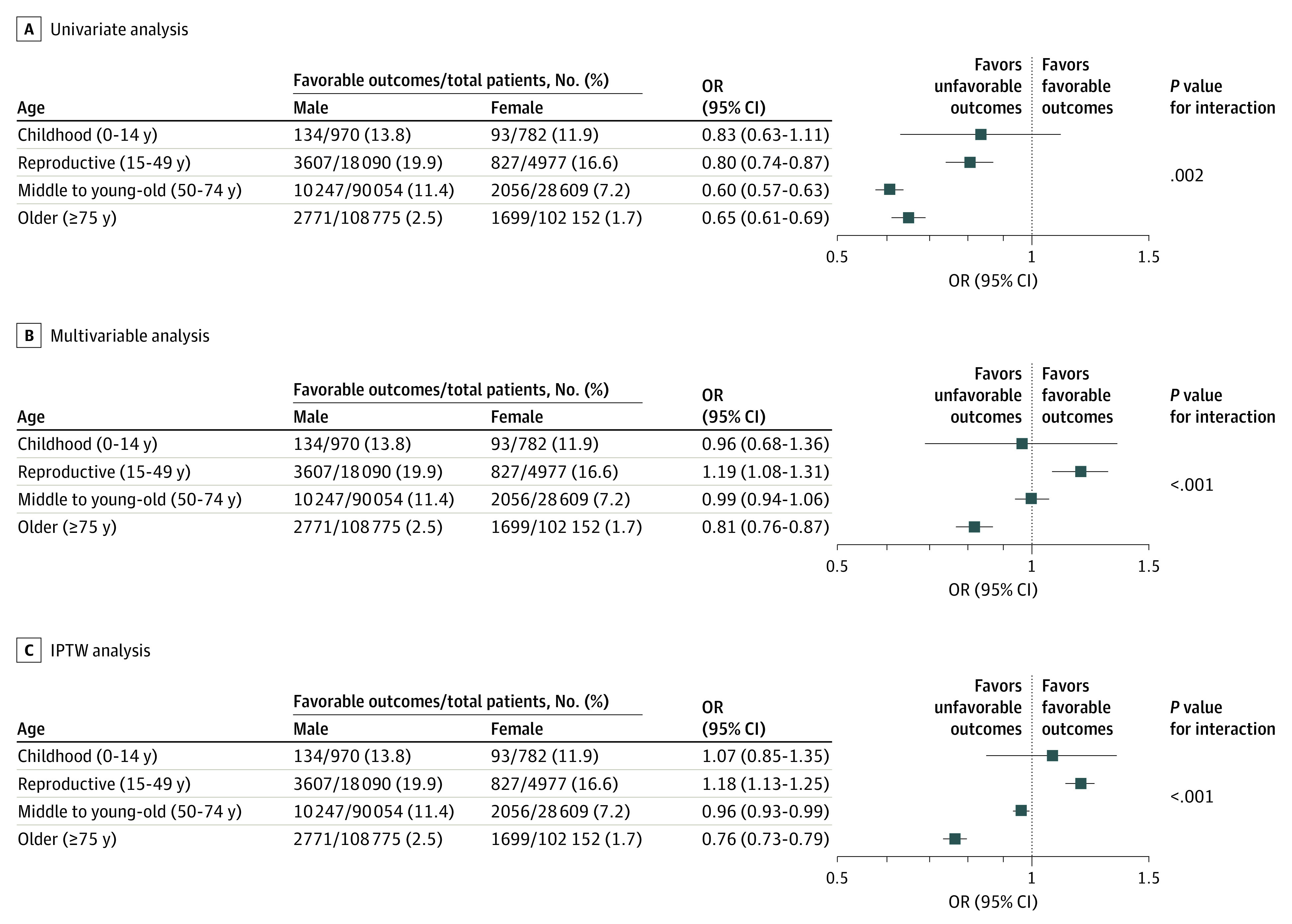
Mixed-Effects Logistic Regression Model for Favorable Neurological Outcomes in the Overall Population of Patients With Bystander-Witnessed Out-of-Hospital Cardiac Arrest Odds ratios (ORs) and 95% CIs for females were calculated using a mixed-effects logistic regression model. Multivariable analysis was adjusted for dispatcher assistance, family member, time of emergency call, bystander cardiopulmonary resuscitation, automated external defibrillation, advanced airway management, initial shockable cardiac arrest rhythm, epinephrine administration, and year of out-of-hospital cardiac arrest. IPTW indicates inverse probability of treatment weighting; OR, odds ratio.

The survival rate at 30 days and neurological outcomes are summarized in eTable 2 in Supplement 1. Figure 3 shows the favorable neurological outcomes in the study population stratified by sex and age. The highest rate of 30-day favorable neurological outcome was observed in females aged 10 to 14 years and males aged 15 to 19 years (39% and 35%), followed by a gradual decrease associated with aging in both sexes. The univariate mixed-effects logistic regression model showed that females had a higher risk of unfavorable neurological outcomes than males in the reproductive (OR, 0.80; 95% CI, 0.74-0.87), middle to young-old (OR, 0.60; 95% CI, 0.57-0.63), and older (OR, 0.65; 95% CI, 0.61-0.69) age groups (Figure 4A). However, after adjusting for confounding factors (eg, EMS-dispatcher assistance, relationship of bystander to patient [family member or nonfamily member], time of emergency call, type of bystander CPR, AED defibrillation, advanced airway management, initial shockable cardiac arrest rhythm, epinephrine administration, and year of OHCA), the multivariable mixed-effects logistic regression model showed that females in the reproductive age group had a higher risk of favorable neurological outcomes (OR, 1.19; 95% CI, 1.08-1.31), and females in the middle to young-old age group had a lower risk of unfavorable neurological outcome (OR, 0.99; 95% CI, 0.94-1.06) compared with males (Figure 4B). Additionally, the multivariable mixed-effects logistic regression model showed that females had a higher risk of unfavorable neurological outcomes compared with males in the older age group (OR, 0.81; 95% CI, 0.76-0.87) but not in childhood (OR, 0.96; 95% CI, 0.68-1.36), which is consistent with the univariate analysis results.

A significant effect modification was observed between age category and sex. The results of IPTW analysis were consistent with the results of the main analysis except that females had a higher risk of unfavorable neurological outcomes compared with males in the middle to young-old age group (OR, 0.96; 95% CI, 0.93-0.99) (Figure 4C).

### Sensitivity Analysis

To confirm the results of the main analysis, we assessed the association of age and sex with 30-day neurological outcomes in patients who experienced OHCA that was witnessed by nonfamily bystanders. eFigure 4 in Supplement 1 shows the favorable neurological outcomes in patients with OHCA that was witnessed by bystanders stratified by sex and age. As a result of the main analysis, the multivariable mixed-effects logistic regression model showed that females in the reproductive age group had favorable neurological outcomes. The results of the IPTW analysis were consistent with the results of the main analysis. Regarding the rate of receiving public access defibrillation and bystander CPR, a pattern similar to that of the overall population was observed in patients with OHCA that was witnessed by bystanders. In contrast, the rate of receiving public access defibrillation was low across all age groups in patients with OHCA that was witnessed by family members (eFigure 5 in Supplement 1).

eFigure 6 in Supplement 1 shows the difference in the proportion of favorable neurological outcomes in females with OHCA witnessed by bystanders based on public access defibrillation and bystander CPR. Multivariable mixed-effects logistic regression analysis revealed that, in the reproductive age group, the performance of public access defibrillation (OR, 3.51; 95% CI, 2.34-5.27) or bystander CPR (OR, 1.62; 95% CI, 1.20-2.22) was associated with a 30-day favorable neurological outcome in females with OHCA that was witnessed by bystanders (eFigure 6 in Supplement 1).

## Discussion

This retrospective cohort study using data from the All-Japan Utstein Registry of the FDMA showed that younger (ie, reproductive age group) females exhibited a more favorable neurological outcome after OHCA compared with their male counterparts of similar age, after controlling for plausible confounding factors. However, the proportion of younger females receiving public access defibrillation and bystander CPR was lower than that of their male counterparts of the same age, as much as those witnessed by nonfamily bystanders. Additionally, implementing public access defibrillation and bystander CPR was associated with favorable neurological outcomes in younger female patients with OHCA. These findings suggest that an improvement in the rate of implementing these lifesaving interventions for younger females experiencing OHCA is anticipated to yield higher rates of favorable neurological outcomes.

This study found disparities in 30-day neurological outcomes based on sex and age in patients with OHCA witnessed by bystanders. Multivariable analysis showed that the 30-day neurological outcomes for females of reproductive age were beneficial, whereas neurological outcomes for those in the older age group were unfavorable. The outcomes for females in the childhood and middle to young-old age groups were comparable with those for males. Despite previous studies on sex-based disparities in outcomes,^20,21,22^ consensus has yet to be established regarding the role of sex in outcomes in patients with OHCA. Nevertheless, studies have demonstrated that female sex may be associated with neutral or advantageous outcomes after OHCA.^20,21,22^ A retrospective study of registry data from the Los Angeles County Emergency Medical Services Agency investigated sex-based differences in survival and neurological outcomes in 5174 patients with OHCA and found that sex was not associated with survival or neurological outcome after OHCA.^20^ A study using the International Cardiac Arrest Registry, including 1667 patients with OHCA in Western countries, reported that female sex was associated with worse survival but not worse neurological outcomes.^21^ The Pan-Asian Resuscitation Outcomes Study, involving 12 sites in 7 Asian countries and a sample of 40 159 individuals who experienced OHCA, revealed a lower rate of return of spontaneous circulation, survival to discharge, and favorable neurological outcomes in univariate analysis; however, no significant sex-based disparities were identified in the multivariate analysis.^22^

While these previous findings aligned with certain aspects of the results of the present research, when discussing sex-based differences in outcomes after OHCA, demographic considerations, particularly age, should be considered. In the subgroup analysis of the Pan-Asian Resuscitation Outcomes Study, female sex in the reproductive age group (18-44 years) was a significant factor in the return of spontaneous circulation at the scene or in the emergency department, and favorable overall performance outcomes were found in the multivariate analysis.^22^ A prospective observational study of 26 940 patients with OHCA in 1 prefecture in Japan found that females aged 13 to 49 years had a significantly higher favorable neurological outcome compared with females of other ages and males.^23^ Therefore, the results of the present study were consistent with those of previous studies, and this study enrolled all individuals who experienced OHCA in Japan, minimizing the potential for selection bias. Furthermore, this study meets the criteria for a natural experiment observational study, strengthening the hypothesis that females of reproductive age had better survival and neurological outcomes.

Moreover, a feature of this study was that the median age of the patients was approximately 10 years older than that in previous studies from Western countries.^20,21^ This discrepancy is reflective of Japan having the highest life expectancy of any country in the world, with 81.47 years’ expectancy for males and 87.57 years’ expectancy for females in 2021.^24^ In the present study, the sex-based disparities in receiving public access defibrillation observed in the younger groups were mitigated in the older group (≥75 years) (Figure 2). Rather, the rate of receiving bystander CPR was significantly higher in females (Figure 2). However, females in the older group had worse neurological outcomes than their male counterparts of the same age, in contrast to the younger groups (Figure 4). This interaction between sex and age on neurological outcome after OHCA was similar to that reported in other studies from Asian countries.^22,23^ Although a plausible mechanism for this interaction is the potential cardiac and neuroprotective properties of endogenous estrogen and progesterone hormones in females after cardiac arrest and resuscitation,^25,26,27,28^ further investigation is needed to elucidate the mechanisms from sociological, pathological, and endocrinological approaches.

The multivariable analysis, which was adjusted for prehospital factors, showed that the neurological outcomes of females of reproductive age were favorable, but these outcomes were found to be unfavorable in the univariate analysis. Public access defibrillation and bystander CPR are the important components of the chain of survival and lifesaving procedures for improving neurological outcome, as shown in eFigure 6 in Supplement 1. However, previous studies have found that females of reproductive age were less likely to have public access defibrillation and bystander CPR than males,^9,22^ concordant with the results of this study (Figure 2). The finding that younger females were less likely to receive lifesaving interventions should be deemed a plausible hypothesis given that this study was free of selection bias.

The barriers to receiving public access defibrillation and bystander CPR among younger females with OHCA should be addressed, and findings of this study suggest that addressing this problem can improve neurological outcomes for this population. These barriers are assumed to be anxiety over the potential allegations of inappropriate touching, sexual assault, or inflicting harm due to the physical vulnerability of female bodies as well as the misconceptions surrounding female persons experiencing medical emergencies.^29^ These factors may prevent bystanders from providing necessary lifesaving interventions to females.^29^ Eliminating these barriers requires legally protecting bystanders’ reasonable lifesaving actions, allowing them to help patients with OHCA without fear of being sued or penalized for mishandling. The Good Samaritan concept should be widely promoted within communities, and public education on resuscitation should be provided.

### Limitations

This study had some limitations. First, no data on cardiac arrest location, comorbidities, medications, activities of daily living, and in-hospital treatment (eg, coronary angiography or targeted temperature management) were included in the registry; these are unmeasured confounding factors that may have biased the study results. Such information is essential for a more precise evaluation of sex- and age-based disparities. Future studies that examine these factors are warranted. Second, given the paucity of information on the demographic characteristics of bystanders, such as age and sex, the demographic composition of those who did not engage in lifesaving interventions remains indeterminate. Third, the registry enrolled all patients with OHCA in Japan; thus, the generalizability of this study’s findings to other nations is uncertain because of variations in EMS systems across different countries. Fourth, the registry did not include a variable of whether an AED had been attached, but it included a variable of whether AED defibrillation had been performed. Therefore, it was not feasible to differentiate between patients who had an AED attached but for whom defibrillation was not indicated and patients who did not have an AED attached. Consequently, it was impossible to determine whether the low incidence of public access defibrillation among younger females was associated with a lower frequency of VF or pulseless VT or a lack of application at the scene.

## Conclusions

This cohort study outlined the current patterns of public access defibrillation and bystander CPR interventions in Japan for patients who experience OHCA, with a focus on age- and sex-based disparities. Results of this study suggest that higher rates of favorable neurological outcomes among patients with OHCA were associated with increased use of public access defibrillation and bystander CPR, particularly in younger females.

## Notes

Supplement 1. **eTable 1.** Baseline Characteristics According to Sex and Age Category **eTable 2.** Outcomes of Patients With Out-of-Hospital Cardiac Arrest According to Sex **eFigure 1.** Study Flowchart According to Age Category **eFigure 2.** Age and Sex Difference in Proportion of VF/Pulseless VT in Patients With No Bystander-CPR **eFigure 3.** Temporal Trends in Rate of Receiving Public-Access Defibrillation and Bystander-CPR According to Sex **eFigure 4.** Sex and Age Difference in Favorable Neurological Outcome When Witnessed by Non-Family Bystanders **eFigure 5.** Age and Sex Difference in Rate of Receiving Public-Access Defibrillation and Bystander-CPR According to Relationship of Bystander to Patient **eFigure 6.** Difference in Proportion of Favorable Neurological Outcome in Female OHCA Witnessed by Non-Family Bystanders According to Public-Access Defibrillation and BCPR

Supplement 2.
Data Sharing Statement
