# Supplementary material for: Sex- and Age-Based Disparities in Public Access Defibrillation, Bystander Cardiopulmonary Resuscitation, and Neurological Outcome in Cardiac Arrest
Source: JAMA Netw Open. 2023 Jul 5;6(7):e2321783. doi: 10.1001/jamanetworkopen.2023.21783 (PMC10323705; doi:10.1001/jamanetworkopen.2023.21783)
Supplement: Supplement 1. — eTable 1. Baseline Characteristics According to Sex and Age Category eTable 2. Outcomes of Patients With Out-of-Hospital Cardiac Arrest According to Sex eFigure 1. Study Flowchart According to Age Category eFigure 2. Age and Sex Difference in Proportion of VF/Pulseless VT in Patients With No Bystander-CPR eFigure 3. Temporal Trends in Rate of Receiving Public-Access Defibrillation and Bystander-CPR According to Sex eFigure 4. Sex and Age Difference in Favorable Neurological Outcome When Witnessed by Non-Family Bystanders eFigure 5. Age and Sex Difference in Rate of Receiving Public-Access Defibrillation and Bystander-CPR According to Relationship of Bystander to Patient eFigure 6. Difference in Proportion of Favorable Neurological Outcome in Female OHCA Witnessed by Non-Family Bystanders According to Public-Access Defibrillation and BCPR [file jamanetwopen-e2321783-s001.pdf]

Supplemental Online Content

Ishii M, Tsujita K, Seki T, et al; Japanese Circulation Society with Resuscitation Science Study (JCS-ReSS) Investigators. Sex- and age-based disparities in public access defibrillation, bystander cardiopulmonary resuscitation, and neurological outcome in cardiac arrest. *JAMA Netw Open*. 2023;6(7):e2321783. doi:10.1001/jamanetworkopen.2023.21783

eTable 1. Baseline Characteristics According to Sex and Age Category
eTable 2. Outcomes of Patients With Out-of-Hospital Cardiac Arrest According to Sex
eFigure 1. Study Flowchart According to Age Category
eFigure 2. Age and Sex Difference in Proportion of VF/Pulseless VT in Patients With No Bystander-CPR
eFigure 3. Temporal Trends in Rate of Receiving Public-Access Defibrillation and Bystander-CPR According to Sex
eFigure 4. Sex and Age Difference in Favorable Neurological Outcome When Witnessed by Non-Family Bystanders
eFigure 5. Age and Sex Difference in Rate of Receiving Public-Access Defibrillation and Bystander-CPR According to Relationship of Bystander to Patient
eFigure 6. Difference in Proportion of Favorable Neurological Outcome in Female OHCA Witnessed by Non-Family Bystanders According to Public-Access Defibrillation and BCPR

This supplemental material has been provided by the authors to give readers additional information about their work.

© 2023 Ishii M et al. *JAMA Network Open.*

eTable 1. Baseline characteristics according to sex and age category

	missing	Childhood (0-14)			Reproductive age (15-49)			Middle to young-old age (50-74)			Old-old age (75-)		
		man	woman	SMD	man	woman	SMD	man	woman	SMD	Man	woman	SMD
n		970	782		18,090	4,977		90,054	28,609		108,775	102,152	
Age, years	0	2 (0, 9)	1 (0, 7)	0.14	42 (35, 46)	42 (35, 46)	0.01	66 (60, 70)	68 (62, 71)	-0.20	83 (79, 87)	87 (82, 91)	-0.55
Relationship of bystander to patient	0												
Family member		719 (74%)	619 (79%)	-0.12	9,394 (52%)	3,363 (68%)	-0.32	54,066 (60%)	20,765 (73%)	-0.27	78,686 (72%)	58,746 (58%)	0.31
Dispatcher-assisted CPR	0	464 (48%)	389 (50%)	-0.04	8,494 (47%)	2,366 (48%)	-0.01	40,884 (45%)	13,228 (46%)	-0.02	53,684 (49%)	50,909 (50%)	-0.01
Type of bystander-initiated CPR	0			0.03			-0.07			-0.04			0.12
Chest compressions only		331 (34%)	292 (37%)		7,626 (42%)	1,932 (39%)		34,674 (39%)	10,356 (36%)		42,614 (39%)	43,625 (43%)	
Chest compressions with rescue breathing		256 (26%)	179 (23%)		2,643 (15%)	731 (15%)		9,259 (10%)	3,076 (11%)		9,697 (8.9%)	13,651 (13%)	
no bystander-initiated CPR		383 (39%)	311 (40%)		7,821 (43%)	2,314 (46%)		46,121 (51%)	15,177 (53%)		56,464 (52%)	44,876 (44%)	
Public-access defibrillation performed by bystander	0	57 (5.9%)	31 (4.0%)	0.09	1,271 (7.0%)	187 (3.8%)	0.15	4,024 (4.5%)	468 (1.6%)	0.17	1,599 (1.5%)	1,396 (1.4%)	0.01
CPR protocol based on Japan Resuscitation Council guidelines	0			-0.04			-0.05			-0.01			0.02
2005		407 (42%)	308 (39%)		6,640 (37%)	1,698 (34%)		33,191 (37%)	10,510 (37%)		32,932 (30%)	31,444 (31%)	
2010		293 (30%)	248 (32%)		5,847 (32%)	1,659 (33%)		28,440 (32%)	8,984 (31%)		36,069 (33%)	34,459 (34%)	
2015		270 (28%)	226 (29%)		5,603 (31%)	1,620 (33%)		28,423 (32%)	9,115 (32%)		39,774 (37%)	36,249 (35%)	
Time between patient collapse and initiation of bystander CPR, mins		1.0 (0.0, 4.0)	1.0 (0.0, 5.0)	-0.03	2.0 (0.0, 5.0)	2.0 (0.0, 5.0)	-0.07	2.0 (0.0, 5.0)	2.0 (0.0, 5.0)	-0.03	2.0 (0.0, 5.0)	1.0 (0.0, 5.0)	0.14
missing	4,536 (1.3%)	25	8		282	73		1,136	334		1,329	1,349	
Time between patient collapse and arrival of emergency medical service, mins		9 (6, 12)	9 (7, 13)	-0.04	9 (6, 12)	9 (6, 13)	-0.07	9 (6, 12)	9 (6, 13)	-0.03	10 (6, 14)	9 (6, 14)	0.06
missing	8,058 (2.3%)	30	23		342	127		1,883	691		2,505	2,457	
Time between placing of emergency call and arrival of emergency medical service, mins		7.0 (5.0, 9.0)	7.0 (5.0, 9.0)	0.01	7.0 (5.0, 9.0)	7.0 (5.0, 9.0)	-0.02	7.0 (5.0, 9.0)	7.0 (6.0, 9.0)	0.02	7.0 (6.0, 9.0)	7.0 (6.0, 9.0)	0.04
missing	344 (0.1%)	1	0		20	6		118	29		85	85	
Advanced airway management		122 (13%)	79 (11%)	0.07	8,007 (46%)	2,124 (44%)	0.03	43,292 (50%)	14,001 (50%)	-0.02	51,483 (49%)	44,531 (45%)	0.08
missing	11,305 (3.2%)	30	35		571	173		2,648	818		3,581	3,449	
Adrenaline	0	24 (2.5%)	18 (2.3%)	0.01	4,366 (24%)	889 (18%)	0.15	23,320 (26%)	6,623 (23%)	0.06	28,349 (26%)	21,764 (21%)	0.11
Initial rhythm	0			-0.11			-0.37			-0.37			-0.19
VF/pulseless VT		189 (19%)	123 (16%)		9,557 (53%)	1,639 (33%)		33,910 (38%)	5,665 (20%)		14,260 (13%)	7,284 (7.1%)	
PEA		272 (28%)	223 (29%)		2,733 (15%)	1,178 (24%)		24,246 (27%)	9,973 (35%)		38,819 (36%)	36,791 (36%)	
Asystole		453 (47%)	377 (48%)		5,281 (29%)	1,983 (40%)		29,799 (33%)	12,359 (43%)		53,688 (49%)	56,136 (55%)	

© 2023 Ishii M et al. *JAMA Network Open.*

Other	56 (5.8%)	59 (7.5%)	519 (2.9%)	177 (3.6%)	2,099 (2.3%)	612 (2.1%)	2,008 (1.8%)	1,941 (1.9%)

Data are n (%), or median (IQR). CPR indicates cardiopulmonary resuscitation, VF; ventricular fibrillation, VT; ventricular tachycardia, PEA; pulseless electrical asystole, SMD; standardized mean difference.

© 2023 Ishii M et al. *JAMA Network Open.*

eTable 2. Outcomes of patients with out-of-hospital cardiac arrest according to sex

	Overall<br>n = 354,409	male<br>n = 217,889	female<br>n = 136,520
CPC, n (%)			
1: good cerebral performance	17,770 (5.0%)	14,004 (6.4%)	3,766 (2.8%)
2: moderate cerebral disability	3,838 (1.1%)	2,878 (1.3%)	960 (0.7%)
3: severe cerebral disability	4,950 (1.4%)	3,559 (1.6%)	1,391 (1.0%)
4: coma or vegetative state	9,113 (2.6%)	6,400 (2.9%)	2,713 (2.0%)
5: death or brain death	318,738 (90%)	191,048 (88%)	127,690 (94%)
Survival at 30 days, n (%)	36,368 (10.3%)	27,273 (12.5%)	9,095 (6.7%)
Survival at 30 days with CPC of 1 or 2, n (%)	21,434 (6.0%)	16,759 (7.7%)	4,675 (3.4%)

CPC indicates Cerebral Performance Category.

© 2023 Ishii M et al. *JAMA Network Open.*

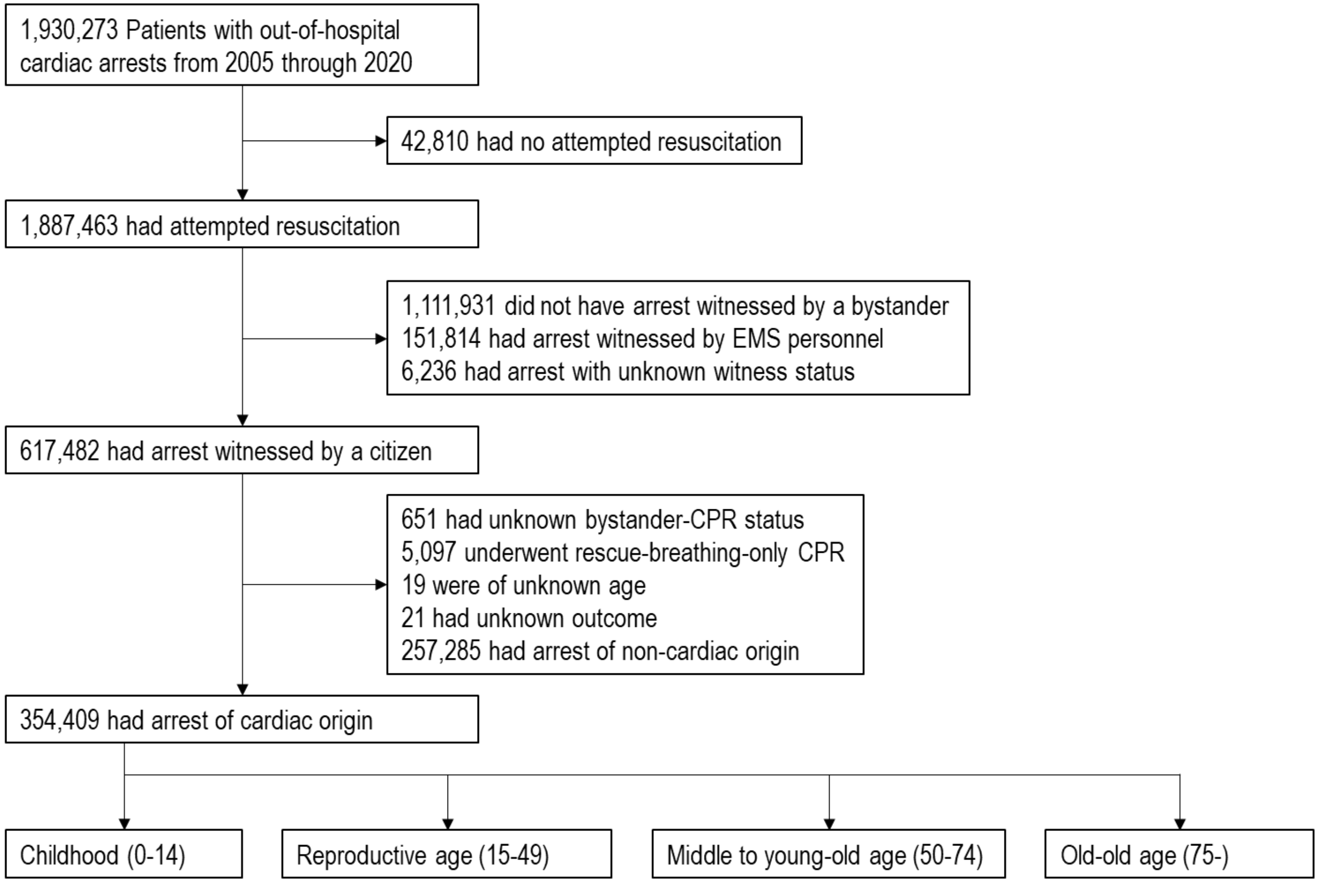

eFigure 1. Study flowchart according to age category.

EMS indicates emergency medical service, CPR; cardiopulmonary resuscitation.

© 2023 Ishii M et al. *JAMA Network Open.*

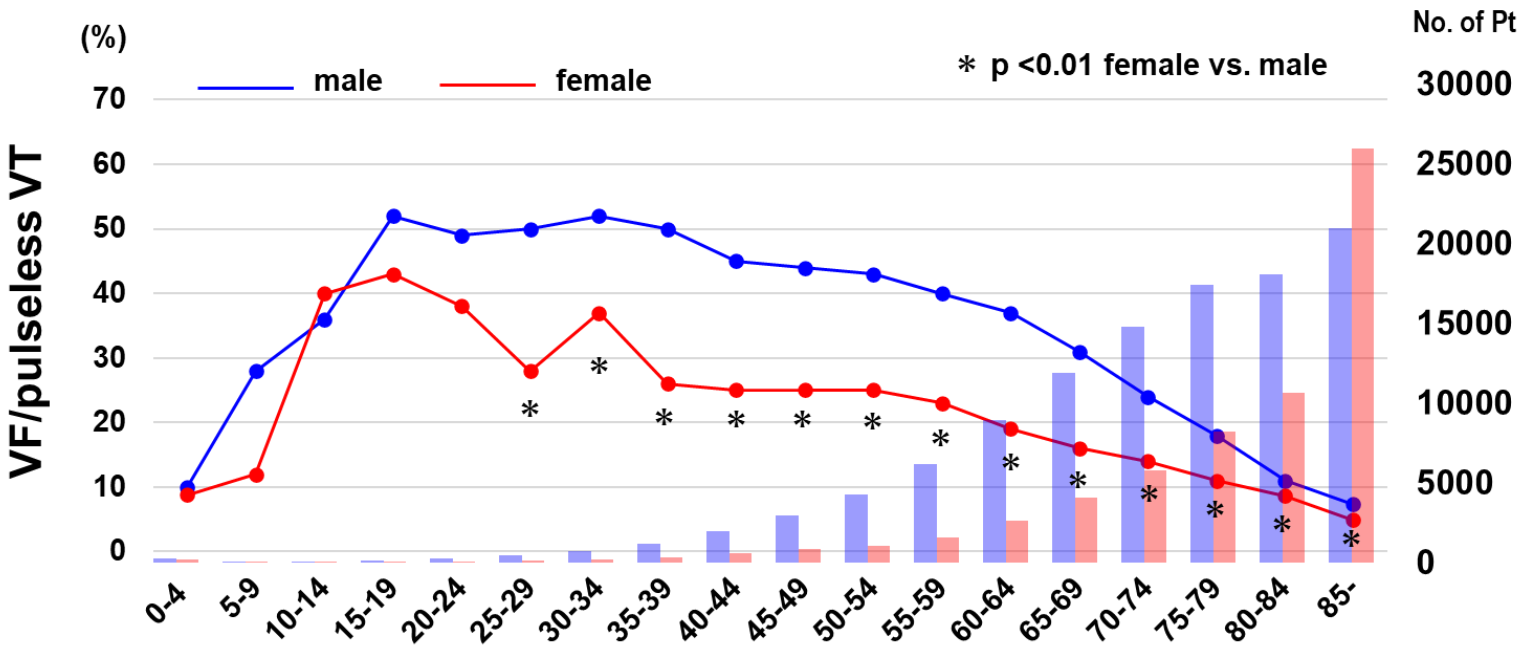

eFigure 2. Age and sex difference in proportion of VF/pulseless VT in patients with no bystander-CPR.

Line graphs show the rate of VF or pulseless VT in patients who did not receive bystander-CPR stratified by sex. Bar graphs show the number of those patients.

VF indicates ventricular fibrillation, VT; ventricular tachycardia.

© 2023 Ishii M et al. *JAMA Network Open.*

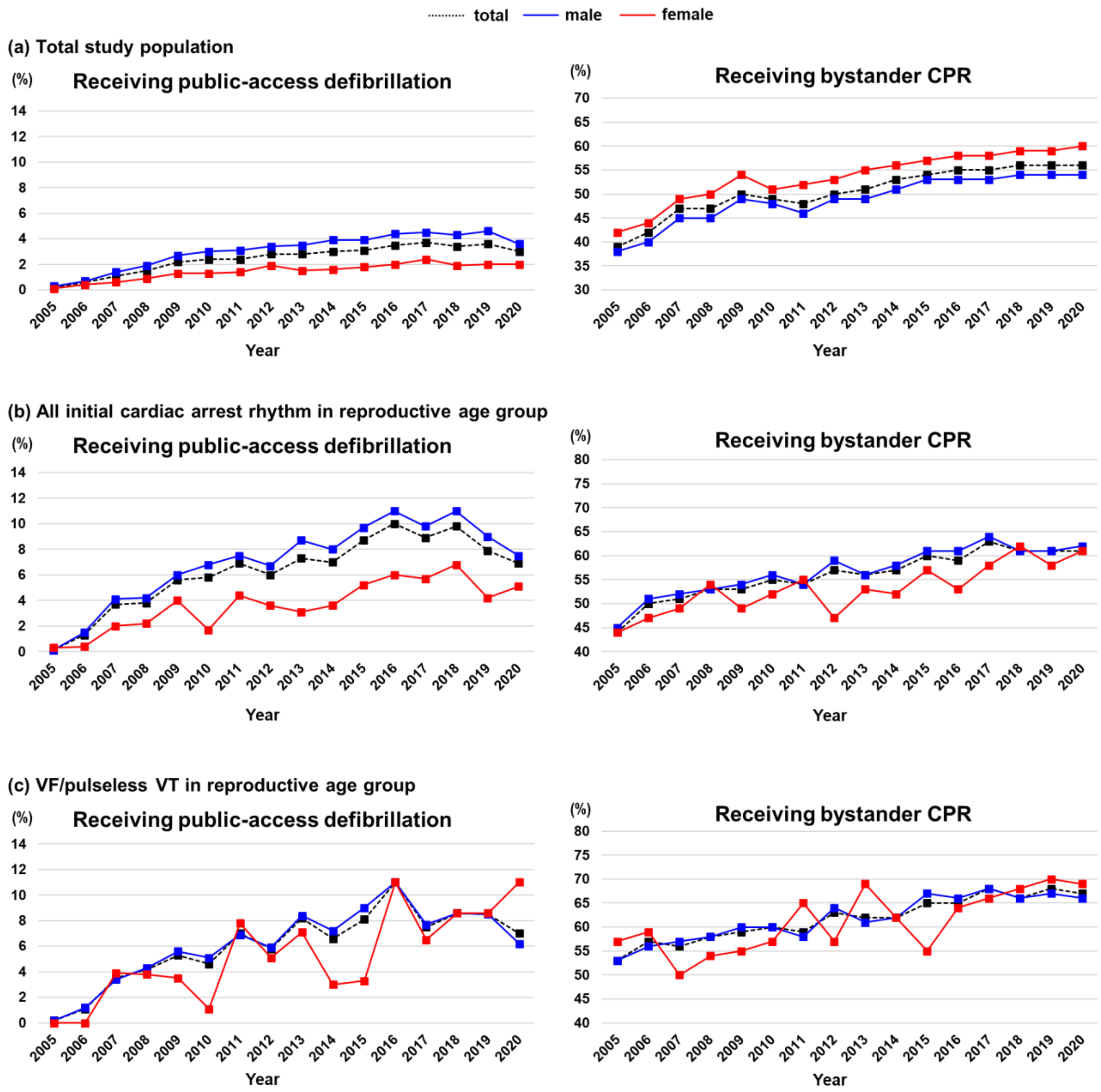

eFigure 3. Temporal trends in rate of receiving public-access defibrillation and bystander-CPR according to sex.

Line graphs show the rate of receiving public-access defibrillation and bystander-CPR stratified by sex in OHCA patients in (a) total study population, (b) the reproductive age group, and (c) patients who have initial VF/pulseless VT in the reproductive age group. CPR indicates cardiopulmonary resuscitation, VF; ventricular fibrillation, VT; ventricular tachycardia

© 2023 Ishii M et al. *JAMA Network Open.*

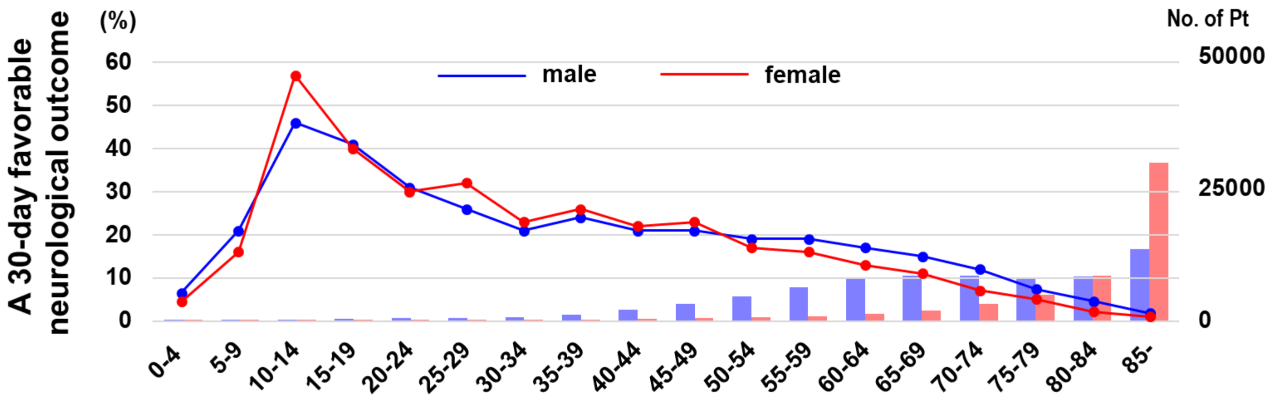

Age	Non-family member: Male no. of interest / no. of patients (%)	Non-family member: Female no. of interest / no. of patients (%)	Multivariable analysis: OR	95%CI		P value	P interaction	IPTW analysis: OR	95%CI		P value	P interaction
							<0.001					<0.001
Childhood (0-14 yr)	71 / 251 (28)	48 / 163 (29)	1.815	1.050	3.138	0. 033		1.541	1.055	2.250	0.025	
Reproductive (15-49 yr)	1,999 / 8,696 (23)	408 / 1,614 (25)	1.308	1.133	1.511	<0.001		1.397	1.303	1.498	<0.001	
Middle to young-old (50-74 yr)	5,690 / 35,988 (16)	838 / 7,844 (11)	0.831	0.761	0.906	<0.001		0.915	0.878	0.953	<0.001	
Old-old (≥75 yr)	1,235 / 30,089 (4.1)	731 / 43,406 (1.7)	0.533	0.482	0.589	<0.001		0.532	0.498	0.569	<0.001	

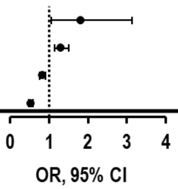

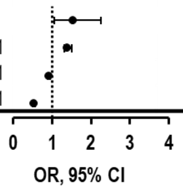

eFigure 4. Sex and age difference in favorable neurological outcome when witnessed by non-family bystanders.

Line graphs show the rate of 30-day favorable neurological outcomes stratified by sex and age in patients who had a bystander-witnessed out-of-hospital cardiac arrest of cardiac origin when witnessed by a non-family member. Bar graphs show the number of patients. Odds ratios and 95% confidence intervals of females for the outcome were calculated using a mixed-effects logistic regression model. Multivariable analysis was adjusted for dispatcher instruction, time of emergency call, BCPR, AED defibrillation, advanced airway management, initial shockable arrest rhythm, adrenaline administration, and year of OHCA.

© 2023 Ishii M et al. *JAMA Network Open.*

OR, odds ratio; CI, confidence interval; BCPR, bystander cardiopulmonary resuscitation; AED, automated external defibrillator.

© 2023 Ishii M et al. *JAMA Network Open.*

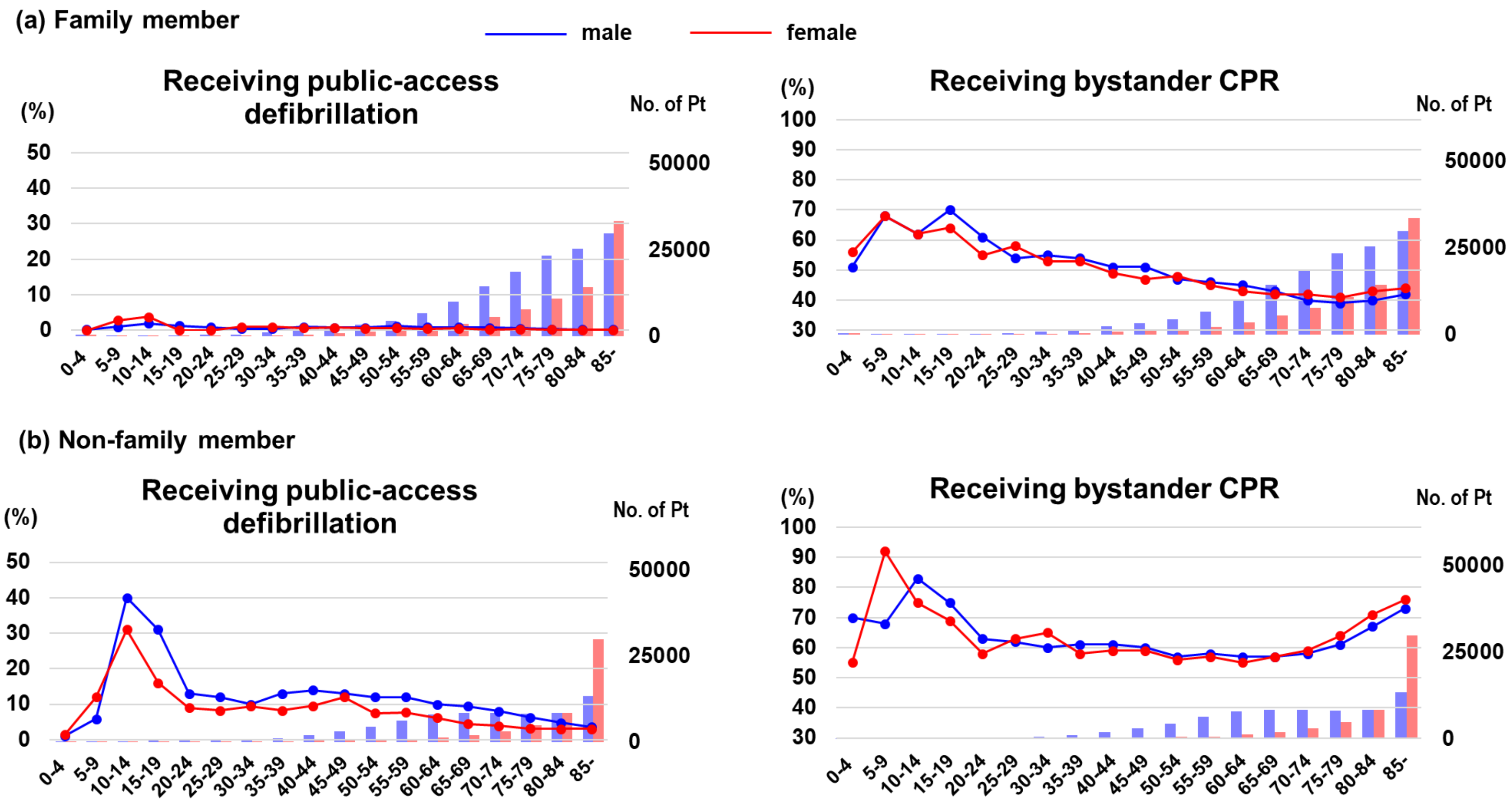

eFigure 5. Age and sex difference in rate of receiving public-access defibrillation and bystander-CPR according to relationship of bystander to patient.

Line graphs show the rate of receiving public-access defibrillation and bystander-CPR stratified by sex in OHCA patients witnessed by (a) family member and (b) non-family member. Bar graphs show the number of those patients. CPR indicates cardiopulmonary resuscitation.

© 2023 Ishii M et al. *JAMA Network Open.*

(a) Public-access defibrillation

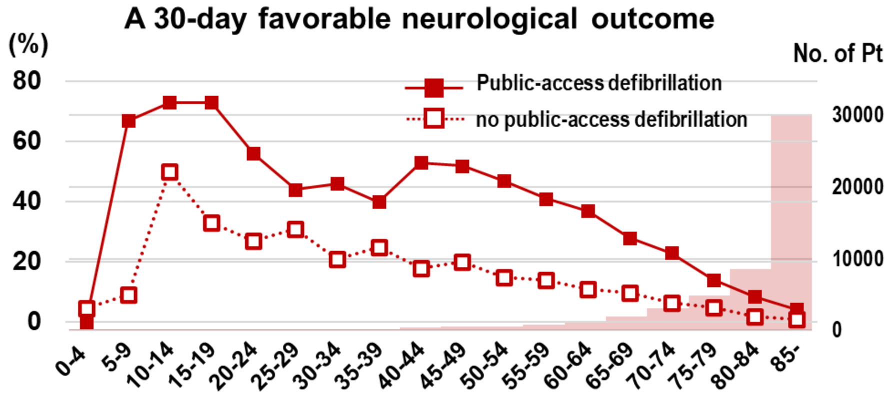

Age	Public-access defibrillation	No public-access defibrillation	Multivariable analysis			
	no. of interest / no. of patients (%)	no. of interest / no. of patients (%)	Adjusted OR	95%CI		P interaction
						0.03
Childhood (0-14 yr)	18/26 (69)	30/137 (22)	10.54	3.136	30.54	
Reproductive (15-49 yr)	86/166 (52)	322/1,448 (22)	3.508	2.338	5.263	
Middle to young-old (50-74 yr)	130/399 (33)	708/7,445 (9.5)	3.581	2.706	4.739	
Old-old (≥75 yr)	81/1,306 (6.2)	650/42,100 (1.5)	3.688	2.811	4.838	

(b) bystander-CPR

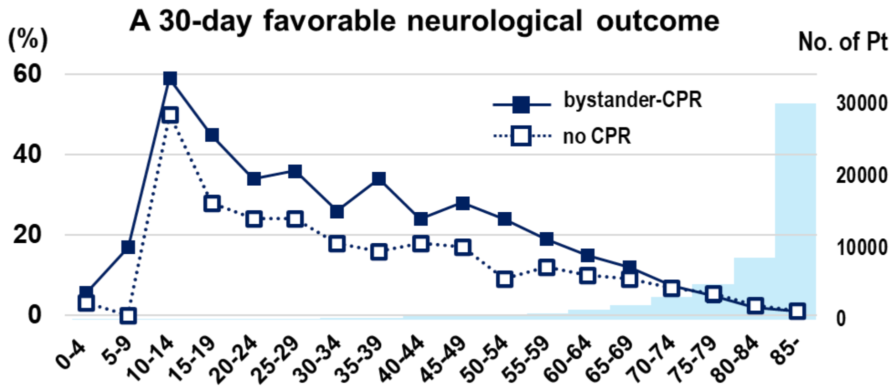

Age	Bystander-CPR	No bystander-CPR	Multivariable analysis			
	no. of interest / no. of patients (%)	no. of interest / no. of patients (%)	Adjusted OR	95%CI		P interaction
						<0.001
Childhood (0-14 yr)	38/113 (34)	10/50 (20)	0.802	0.283	2.272	
Reproductive (15-49 yr)	290/971 (30)	118/643 (18)	1.623	1.197	2.220	
Middle to young-old (50-74 yr)	546/4,506 (12)	292/3,338 (8.7)	1.215	1.010	1.462	
Old-old (≥75 yr)	497/32,084 (1.5)	234/11,322 (2.1)	0.774	0.649	0.923	

eFigure 6. Difference in proportion of favorable neurological outcome in female OHCA witnessed by non-family bystanders according to public-access defibrillation and BCPR.

© 2023 Ishii M et al. *JAMA Network Open.*

Line graphs show the rate of 30-day favorable neurological outcomes stratified by age in female patients with OHCA with and without (a) public-access defibrillation or (b) BCPR. Bar graphs show the number of patients. Odds ratios and the 95% confidence intervals for the outcomes were calculated using a mixed-effects logistic regression model. Multivariate analysis included variables such as dispatcher instruction, time of emergency call, BCPR, AED defibrillation, advanced airway management, initial shockable arrest rhythm, adrenaline administration, and year.

OR, odds ratio; CI, confidence interval; BCPR, bystander cardiopulmonary resuscitation; AED, automated external defibrillator.

© 2023 Ishii M et al. *JAMA Network Open.*
