# Supplementary material for: Sex- and Age-Based Disparities in Public Access Defibrillation, Bystander Cardiopulmonary Resuscitation, and Neurological Outcome in Cardiac Arrest
Source: JAMA Netw Open. 2023 Jul 5;6(7):e2321783. doi: 10.1001/jamanetworkopen.2023.21783 (PMC10323705; doi:10.1001/jamanetworkopen.2023.21783)
Supplement: Supplement 2. — Data Sharing Statement [file jamanetwopen-e2321783-s002.pdf]

Data Sharing Statement

Ishii. Sex- and Age-Based Disparities in Public Access Defibrillation, Bystander Cardiopulmonary Resuscitation, and Neurological Outcome in Cardiac Arrest. *JAMA Netw Open*. Published July 05, 2023. doi:10.1001/jamanetworkopen.2023.21783

Data
Data available: No
